# Defined serum‐free three‐dimensional culture of umbilical cord‐derived mesenchymal stem cells yields exosomes that promote fibroblast proliferation and migration in vitro

**DOI:** 10.1096/fj.202001768RR

**Published:** 2020-12-25

**Authors:** Farid N. Faruqu, Revadee Liam‐Or, Shuai Zhou, Rebecca Nip, Khuloud T. Al‐Jamal

**Affiliations:** ^1^ Institute of Pharmaceutical Science King’s College London London UK

**Keywords:** Aggrewell plates, fibroblasts, platelet lysate, proliferation, wound healing

## Abstract

Stem cell‐derived exosomes are emerging as novel and clinically relevant cell‐free therapeutics for regenerative therapy. This work focused on investigating the stimulation of fibroblasts by exosomes derived from umbilical cord‐derived mesenchymal stem cells (ucMSC) in a defined serum‐free three‐dimensional (3D) culture. 3D culture of ucMSC was carried out in medium supplemented with KnockOut serum replacement (KO‐medium) using the Aggrewell system. ucMSC in KO‐medium formed spheroids with maintained size and integrity throughout culture. This enabled the isolation of vesicles from ucMSC spheroids in KO‐medium with sizes that fall within the exosomal size range and were positive for the expression of canonical exosomal markers CD63, CD9, CD81, Alix, and TSG101. The ucMSC‐derived exosomes (Exo_ucMSC_) were shown to significantly increase the migration and proliferation of murine fibroblasts in vitro. To conclude, 3D culture of ucMSC in defined serum‐free KO‐medium formed viable spheroids which enabled the isolation of Exo_ucMSC_ with the potential of accelerating wound healing.

**Abbreviations**
AD: adipose
AKT: protein kinase B
BCA: bicinchoninic acid
BM: bone marrow
CANX: calnexin
CM: conditioned medium
CXCR4: C‐X‐C chemokine receptor type 4, EGF, epidermal growth factor
EV: extracellular vesicles
ECM: extracellular matrix
ED‐HS: exosome‐depleted human serum
ESCRT: endosomal sorting complexes required for transport
FBS: fetal bovine serum
GCCP: good cell culture practice
GMP: good manufacturing practice
HGF: hepatocyte growth factor
HDF: human dermal fibroblasts
HLA‐DR: human leukocyte antigen—DR isotype
hPL: human platelet lysate
HS: human serum
KO: KnockOut serum replacement
MTT: 3‐(4,5‐dimethylthiazol‐2‐yl)‐2,5‐diphenyltetrazolium bromide
NTA: nanoparticle tracking analysis
siRNA: small interfering ribonucleic acid, TBS, tris‐buffered saline
TGFβ: transforming growth factor beta
TLR: toll‐like receptors
TSG101: tumor susceptibility gene 101
ucMSC: umbilical cord‐derived mesenchymal stem cells
UVB: ultraviolet B

## 1 INTRODUCTION

Mesenchymal stem cells (MSC) are a type of adult human stem cells residing in various tissues in the body and function mainly in repair following trauma, disease, or aging.^1^ MSC are minimally defined as plastic‐adherent cells capable of tri‐lineage (bone, cartilage, and fat) differentiation which are CD105^+^ CD73^+^ CD90^+^ and CD45^–^ CD34^–^ CD11b^–^ CD14^–^ CD79a^–^HLA‐DR^–^.^2^ MSC were reported to show therapeutic effects such as repair of damaged or injured tissues and suppression of pathological inflammation.^3^ Combined with advantages such as ethical cell sourcing, ease of isolation and in vitro expansion, and low immunogenicity, MSC represent the most widely used stem cell in therapy with over 1,000 ongoing clinical studies.^4^ Although MSC are more commonly sourced from bone marrow and adipose tissue, umbilical cord‐derived MSC (ucMSC) are gaining popularity as a source of MSC for therapy.^5^ Among advantages offered by ucMSC include availability of umbilical cords as medical waste, not involving painful invasive procedures as well as having higher proliferation, differentiation, and transfection efficiency compared to MSC from other sources.^5^, ^6^

Therapeutic effects of MSC are largely mediated by the paracrine secretion of an array of bioactive components (the secretome), which consists of soluble factors and extracellular vesicles (EV),^7^ with the latter more implicated in mediating these effects.^8^ Exosomes are a subtype of EV secreted by various cell types,^9^, ^10^ structurally bound by a phospholipid bilayer membrane enclosing an aqueous core, with a typical hydrodynamic size range between 50 and 200 nm in diameter.^11^ Exosomes inherently carry biomolecules such as proteins^12^, ^13^ and nucleic acids^14^, ^15^ where they play a role in intercellular communications by delivering these cargoes, eliciting functional outcomes upon uptake and processing by recipient cells. Exosomes are distinguished from other EV subtypes by their endosomal origin, and are defined by the positive expression of ESCRT‐related proteins such as Alix and TSG101, as well as the tetraspanins CD63, CD9, and CD81.^16^ MSC‐derived exosomes (MSC‐Exo) has been reported to exhibit therapeutic effects of similar potency to that of MSC themselves, and have been demonstrated to promote regeneration in numerous animal models of lung, brain, heart, and liver injuries.^17^, ^18^ MSC‐Exo is now emerging as novel cell‐free therapeutics for regenerative medicine with at least six studies in clinical trials, as they confer advantages over MSC‐based therapy such as lower risk of lung embolism, tumor formation and undesired tissue ossification upon administration, ease of long‐term storage and logistics, as well as better systemic distribution and tissue penetration (e.g., ability to cross the blood‐brain barrier).^4^, ^19^

Given the advantages of ucMSC for clinical use, the past decade has seen a rapid increase in the number of preclinical studies on the regenerative and therapeutic properties of exosomes derived from ucMSC (Exo_ucMSC_). Exo_ucMSC_ has been reported to alleviate the injuries and disorders of vital organs such as liver,^20^, ^21^ heart,^22^, ^23^ and kidneys.^24^ Exo_ucMSC_ were also reported to inhibit undesired immune responses such as in the context of organ transplantation and burn‐induced inflammation.^25^, ^26^ Among the reported regenerative effects of Exo_ucMSC_ include improved recovery in cutaneous wound healing,^27^, ^28^ endometrial injury,^29^ and UVB‐induced photoaging.^30^

Unfortunately, these studies used FBS in their ucMSC culture, which harbors risks for clinical translation of stem cell‐based therapy such as harmful xenogeneic immune responses, either by internalization and subsequent antigen presentation, or by attachment to cell surface, and therefore, becomes antigenic substrates following transplantation.^31^, ^32^ Other concerns of FBS contamination in administering cells for therapeutic applications include risks of viral and prion transmission,^33^ as well as zoonotic infections from metabolic incorporation of xenogeneic sialic acid Neu5Gc.^32^ Although these risks are associated with therapy involving actual cells, they also apply on the derived exosomes as their membranes mirror that of the parent cells,^16^ and therefore, may also present these antigenic substances. FBS also suffers from batch‐to‐batch variations, subsequently resulting in phenotypic, and therefore, therapeutic variations in the cultured MSCs.^34^ This is where the use of defined serum replacements is important for consistent and xenobiotic‐free medium supplementation, thereby facilitating the achievement of clinical quality cell‐derived samples, compliant with the proposed Good Cell Culture Practice (GCCP) and Good Manufacturing Practice (GMP).^35^

Three‐dimensional (3D) culture of cells enables better recapitulation of the in vivo environment^36^ and was proven to be beneficial for MSC culture in terms of enhancing their therapeutic properties. 3D culture of stem cells was reported to enhance their potency in terms of immunomodulation, angiogenesis, anti‐fibrosis, and differentiation potential.^37^ Such 3D culture has started to be implemented in ucMSC culture, and it was shown to improve their “stemness” and multipotency characteristics.^38^ Expression of CXCR4 which is important for MSC homing during injury, and Toll‐like receptors (TLR) which influence their immunomodulatory properties were also improved in 3D culture compared to 2D culture.^39^ One would hypothesize that the augmentation of therapeutic effects of ucMSC from 3D culture would also be translated to their exosomes, and indeed it has been demonstrated that Exo_ucMSC_ derived from 3D culture showed significantly higher osteochondral regeneration compared to that derived from 2D culture.^19^

Only three studies to date involved culturing ucMSC in 3D format, and two of them used FBS as their ucMSC culture supplementation, including the study that showed enhanced potency of by Exo_ucMSC_ derived from 3D culture compared to that from 2D culture.^19^, ^39^ The third study reported a defined serum‐free media for 3D culture ucMSC but did not extend the work to isolate Exo_ucMSC_ from these cultures nor investigated their therapeutic potential.^38^ The 3D culturing method employed in these studies utilized porcine acellular dermal matrix and hollow fiber bioreactors which did not form proper spheroids for the former nor permitted spheroid growth monitoring for the latter.^19^, ^39^ Additionally, the use of suspension Rocker system requires substantial space in tissue culture incubators which may present as challenge for large scale production.^38^ The present work, therefore, aims to utilize a simple 3D culture method in a defined serum‐free medium using the micropatterned Aggrewell culture plates, for the isolation of Exo_ucMSC_ and subsequent investigation of their regenerative potential in terms of fibroblast stimulation in vitro for accelerated wound healing.

## 2 MATERIALS AND METHODS

### 2.1 Preparation of media for ucMSC culture

The base medium for ucMSC culture consists of MEM‐α supplemented with 1% penicillin/streptomycin and 1% GlutaMax (Thermo Fisher Scientific, UK). Various media were then made by differentially supplementing the base medium as follows: (i) FBS‐supplemented medium (FBS‐medium)—base medium supplemented with 10% FBS (Thermo Fisher Scientific, UK); (ii) Human platelet lysate‐supplemented medium (hPL‐medium)—base medium supplemented with 5% human platelet lysate (Cook Regentec, USA); (iii) KnockOut serum replacement‐supplemented medium (KO‐medium)—base medium supplemented with 20% KnockOut serum replacement (Thermo Fisher Scientific, UK), 1% MEM nonessential amino acids (Sigma‐Aldrich, Dorset, UK), and 0.1 mM β‐mercaptoethanol.

### 2.2 2D culture of umbilical cord‐derived mesenchymal stem cells (ucMSC)

ucMSC were kindly provided by Prof. Francesco Dazzi (Comprehensive Cancer Center, King's College London) and cultured with slight adaptations from previous a report.^40^ Briefly, cells were initially thawed in FBS‐medium (in 175 cm^2^ flasks). The medium was discarded on the following day and replaced with hPL‐medium henceforth. ucMSC were passaged when they reach 80%‐90% following conventional trypsinization protocol, with a minimum of 2 x 10^6^ cells seeded in a flask.

### 2.3 3D culture of ucMSC

This was carried out in Aggrewell 400 plates (STEMCELL Technologies, France). The Aggrewell plates were first incubated in anti‐adherence rinsing solution (500 µL/well—STEMCELL Technologies, France) for 30 minutes—2 hours, during which the ucMSC were harvested from 2D culture. After incubation, the Aggrewell plate was washed with PBS, followed by either hPL‐ or KO‐medium (500 µL/well). Fresh hPL‐ or KO‐medium containing ucMSC at a density of 1.2 × 10^5^ cells/mL/well were added to each well of the Aggrewell plate and mixed thoroughly by pipetting. The plate was centrifuged to collect the cells at the bottom of the microwells and kept in the incubator undisturbed for at least 3 days. The medium was changed after 3 days, during which the cells were imaged. The medium was then changed every 2‐3 days and the culture was maintained for 24 days. Conditioned media (CM) were collected and stored at 4°C for subsequent exosome isolation. ucMSC spheroids were harvested by thorough pipetting using 1 mL pipette tips (ends cut to provide larger orifice) to resuspend the spheroids.

### 2.4 Spheroid size analysis

Size of ucMSC spheroids was measured using ImageJ software (NIH, USA) on images captured by brightfield microscopy at 10X magnification throughout culture. Results were expressed as mean ± SD, n = 15‐30 per condition.

### 2.5 Exosome isolation from conditioned‐medium (CM)

CM was first precleared via filtration through 0.22 µm filters (Merck, UK) and added to polycarbonate ultracentrifuge tubes (25 mL/tube—Beckman Coulter, UK). A sucrose cushion (3 mL/tube, 25% w/w in D_2_O) was added below the CM, and the tubes were centrifuged in a swing‐out rotor at 100,000 g for 1.5 hours at 4°C. The sucrose cushion was withdrawn (2 mL/tube), added to 20 mL filtered PBS, filtered (0.22 µm) into polycarbonate ultracentrifuge bottles (Beckman Coulter, UK), and centrifuged again under similar conditions in a fixed‐angle rotor. The pellet obtained (i.e., the exosomes) was resuspended in 400 µL filtered PBS. ucMSC‐derived exosomes (Exo_ucMSC_) were aliquoted and kept at 4°C and −80°C for short‐ and long‐term storage, respectively.

### 2.6 Nanoparticle tracking analysis (NTA)

The hydrodynamic size and yield of isolated exosomes were measured by nanoparticle tracking analysis (NTA) using NanoSight LM10 (Malvern Instruments, UK). Exosome samples were first diluted in filtered PBS to obtain 20‐80 particles in the viewing frame, and four measurements were done for each sample, with 30 seconds as the duration for each recording. The temperature for each recording was measured and noted in the software. Results were analyzed using the NanoSight NTA 3.2 software (Malvern Instruments, UK) and were expressed as mean ± standard deviation (SD).

### 2.7 Protein measurements and particle‐to‐protein (P:P) ratio calculations

Protein measurements were done using the Micro BCA protein assay kit (Thermo Fisher Scientific, UK) with slight modifications from supplier's instructions. Exosome samples diluted in PBS (1:1), BSA standards also prepared in PBS (500‐3.9 µg/mL, 1:2 serial dilutions) and PBS only as blank were added in triplicates in a 96‐well plate (40 µL/well). BCA reagent prepared according to supplier's instructions was then added to each sample/standard replicate (50 µL/well) then incubated at 37°C for 30 minutes. The absorbance at 562 nm for each sample/standard was measured using FLUOStar Omega plate reader (BMG LabTech, UK). Calculation of protein concentrations was done using the built‐in wizard of the MARS software (BMG LabTech, UK). P:P ratio was then calculated by the dividing the particle concentrations obtained from NTA above by the protein concentrations obtained from the Micro BCA assay.

### 2.8 Detection of exosomal markers by dot blot

Equal amount of proteins (0.5 µg) were spotted on a nitrocellulose membrane (Bio‐Rad, UK), followed by blocking for 1 hour at RT in 3% milk prepared in Tris‐buffered saline (TBS) with 0.1% Tween‐20 (TBS‐T) and incubation with primary antibodies (CD9, CD63, CD81, Alix, TSG101 and CANX) in the blocking buffer overnight at 4°C. The membrane was then washed three times with TBS‐T and incubated with HRP‐conjugated secondary antibody in the blocking buffer for 1 hour at RT. The membrane was washed again as above, and the signals were developed with SuperSignal West Femto Maximum Sensitivity Substrate (Thermo Fisher Scientific, UK), followed by imaging using the Gel Doc system (Bio‐Rad, UK). Images obtained were analyzed using the Image Lab software (Bio‐Rad, UK).

### 2.9 In vitro migration assay (scratch assay)

3T3 cells (murine fibroblasts) were cultured in DMEM (Sigma‐Aldrich, UK) supplemented with 1% penicillin/streptomycin, 1% GlutaMAX, and 10% FBS (Thermo Fisher Scientific, UK). Cells were seeded at 1.2 × 10^5^ cells/well in a 24‐well plate and left overnight to reach full confluency. Cells were then treated with 10 μg/mL mitomycin (Fisher Scientific, UK) in serum‐free medium for 2 hours to inhibit proliferation. Following this, a vertical scratch was made on the confluent cell monolayer with a sterile 1000 μl pipette tip, washed with PBS to remove detached cells and replaced with medium supplemented with 1% FBS, again to inhibit cell proliferation. Cells were then treated with either 10% hPL (positive control) or 10^5^‐10^8^ ucMSC‐derived exosomes (Exo_ucMSC_), of which the treatment volumes were kept constant (50 µL). The control group was treated with 50 µL PBS to account for PBS dilution. Images of the wound area were taken at 0, 24, 48, and 72 hours post‐treatment, and the wound area at each timepoint was measured using ImageJ software (NIH, USA). Rate of fibroblast migration was assessed by the amount of wound area reduction at each timepoint, relative to that at 0 hour post‐treatment, and was calculated as follows:

$$\text{Wound}\;\text{area}\;\text{reduction}\;\left(\%\right)=\frac{{\text{Area}}_{T‐0}‐{\text{Area}}_{T‐1}}{{\text{Area}}_{T‐0}}\times 100.$$

where *T*–0 is 0 hour; and *T*–1 is either 24, 48, or 72 hours.

### 2.10 In vitro proliferation assay (MTT)

3T3 cells were seeded at a density of 1000 cells/well in 96‐well plates and left to settle overnight. Cells were then treated with either 100 ng/mL murine EGF (PeproTech, UK), as a positive control, or 10^5^‐10^8^ Exo_ucMSC_ and incubated for 24, 48, and 72 hours. At each time point, the old medium was discarded and replaced with medium containing 1 μg/mL MTT and left to incubate for 4 hours. The MTT‐containing medium was then removed, and 200 µL DMSO were added to each well, and mixed thoroughly to evenly solubilize the formazan crystals formed. The absorbance at 570 nm was measured using FLUOStar Omega plate reader (BMG LabTech, UK) and the results were analyzed using MARS software (BMG LabTech, UK). The absorbance values of each treatment groups were normalized to that of untreated cells and were taken as the measure of fibroblast proliferation.

### 2.11 Statistical analyses

For all experiments, data were presented as mean ± SD, where *n* denotes the number of repeats. Unless stated otherwise in the respective figure captions, statistical significance of the data was assessed using one‐way ANOVA accompanied by Dunnett's post hoc test for multiple comparisons. Asterisk(s) were used to designate significance as follows: *P < .05, **P < .01, and ***P < .001. Statistical analyses were done using GraphPad Prism 8.4.2 software.

## 3 RESULTS

### 3.1 ucMSC form physically stable spheroids in KO‐medium throughout culture

**FIGURE 1:**
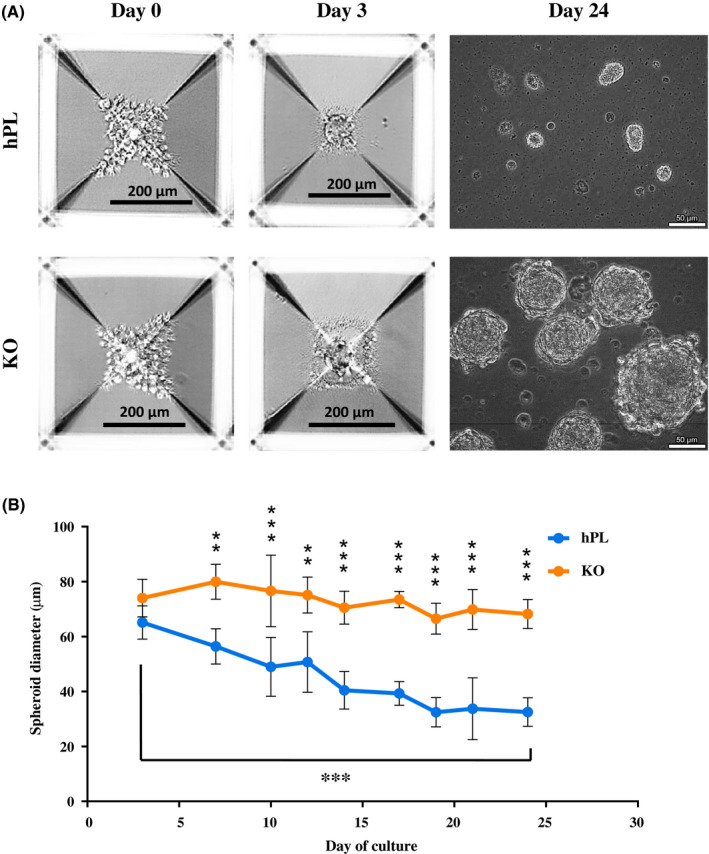
Three‐dimensional (3D) culture of umbilical cord‐derived mesenchymal stem cells (ucMSC) in Aggrewell plates. A, Images show representative morphology of ucMSC forming spheroids in the microwells of the Aggrewell plates in hPL‐ and KO‐supplemented medium by bright field microscopy under 10X magnification throughout culture. Image on Day 24 shows harvested spheroids in suspension under 40X magnification. ucMSC were seeded at a density of 1.2 × 10^5^ cells/well (100 cells/microwell) of the Aggrewell plate. B, Size analysis of ucMSC spheroids in hPL‐ and KO‐medium throughout culture. ucMSC spheroid images taken by brightfield microscopy on each day the media was changed (every 2‐3 days) were analyzed using ImageJ software. Values are expressed as mean ± SD (n = 15‐30). Two‐way ANOVA with Sidak's test for multiple comparisons was used for statistical analysis (*P < .05, **P < .01, ***P < .001)

Three‐dimensional (3D) culture of ucMSC was carried out using the Aggrewell system in both the default culture medium, that is, hPL‐medium and KO‐medium as the defined serum‐free medium. Visual observation by brightfield microscopy showed that ucMSC formed viable spheroids in their default hPL‐medium, and that the spheroids started to form from as early as day 3 of culture (Figure 1A). Similarly, ucMSC also formed viable spheroids in KO‐medium from day 3. ucMSC spheroids in both media maintained their physical integrity until day 24 of culture. Quantitative analysis showed that ucMSC spheroids in both media recorded similar sizes (~70 µm) when they started to form on day 3 of culture (Figure 1B). ucMSC spheroids in hPL‐medium started to become significantly smaller than those in KO‐medium on day 7, and the former continued to show a downward trend to a significantly smaller size of ~33 µm on day 24, relative to that on day 3. ucMSC spheroids in KO‐medium, however, maintained their size of around ~70 µm throughout culture. Representative images of ucMSC spheroids in both media throughout culture are shown in Supplementary Figures 1 and 2 for qualitative reference. In summary, KO‐medium provides a viable defined serum‐free culture condition for 3D culture of ucMSC.

### 3.2 KO‐medium enables isolation of ucMSC‐derived exosomes (Exo_ucMSC_)

**FIGURE 2:**
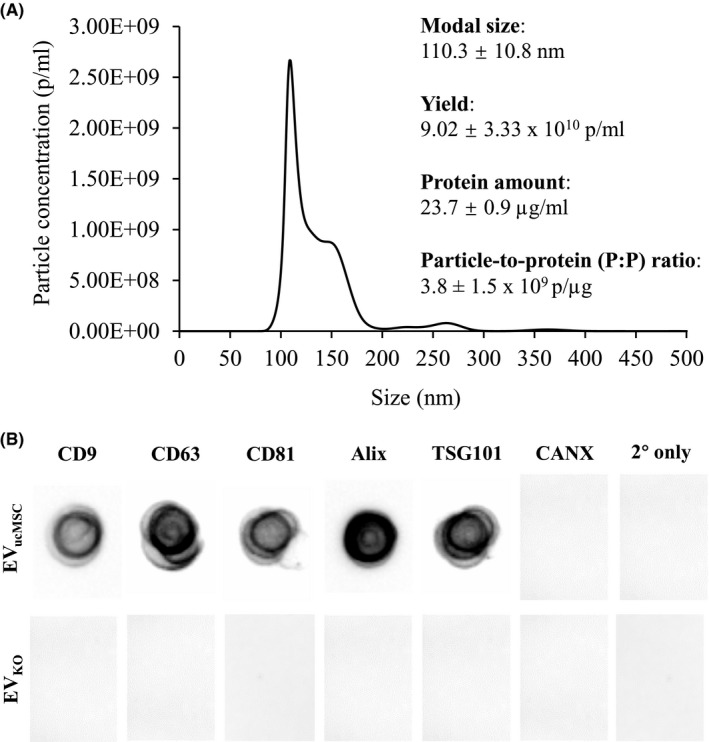
Characterization of EVs isolated from ucMSC (EV_ucMSC_). The EVs were isolated from ucMSC spheroids conditioned medium (CM) within 12 days of culture. A, Physicochemical characterization of EV_ucMSC_. Histogram shows results from nanoparticle tracking analysis (NTA) of EV_ucMSC_, resuspended in 400 µL PBS postisolation. B, Detection of exosomal surface markers CD9, CD63, and CD81; luminal markers Alix and TSG101; and endoplasmic reticulum‐associated protein Calnexin (CANX) on EV_ucMSC_ by dot blot. Equal amounts protein from EV_ucMSC_ (0.5 µg) were spotted on the nitrocellulose membrane prior to staining. The same markers were also analyzed on equal number of “EV” derived from unconditioned KO‐medium subjected to the same exosome isolation protocol as the conditioned media above (EV_KO_—80 µL from 0.5 × 10^10^ p/mL stock). Samples were also stained with secondary antibodies only as control for nonspecific background signals

Exosome isolation was performed on CM collected from ucMSC spheroids in KO‐medium from day 1‐12, as we previously reported that CM collected from Day 13‐24 resulted in exosomes sample of lower quality in terms of contaminating non‐exosomal vesicles.^41^ Nanoparticle tracking analysis (NTA) showed that the EVs isolated from ucMSC spheroids (EV_ucMSC_) in KO‐medium recorded a yield of ~9 × 10^10^ particles/mL, with a modal size of ~110 nm, which falls within the defined exosome size range (Figure 2A). The EV_ucMSC_ sample recorded a protein content of 23.7 ± 0.9 µg/mL. From this, the particle‐to‐protein (P:P) ratio was calculated as a measure of purity from contaminating CM proteins during isolation and was found to be 3.8 ± 1.5 × 10^9^ p/µg, which falls within the proposed medium‐high purity range.^42^ Dot blot analysis revealed that the EV_ucMSC_ were positive for the expression of canonical exosomal markers such as the tetraspanins CD9, CD63, and CD81, as well as endosome‐associated proteins Alix and TSG101, confirming their endosomal origin during biogenesis (Figure 2B). The EV_ucMSC_ were also negative for the expression of endoplasmic reticulum‐associated protein calnexin (CANX), suggesting minimal contamination from non‐exosomal vesicles. Unconditioned KO‐medium (fresh KO‐medium not yet used for cell culture) was also subjected to the same exosome isolation attempt to assess for background particles/vesicles in the medium. Although NTA were able to measure particles/vesicles isolated from unconditioned KO‐medium (EV_KO_), the amount was ~18‐fold lower (~0.5 × 10^10^ p/mL), and particle size was significantly bigger (~173 nm) than that of the EV_ucMSC_ sample (Supplementary Figure 3). Dot blot analysis of EV_KO_ also showed negative expression for all the markers, further suggesting minimal contamination of non‐exosomal vesicles (Figure 2B). In summary, KO‐medium is a suitable defined serum‐free medium for the isolation of exosomes from 3D culture of ucMSC (Exo_ucMSC_) with high purity for subsequent downstream investigations.

### 3.3 Exo_ucMSC_ stimulate fibroblast migration following injury in vitro

**FIGURE 3:**
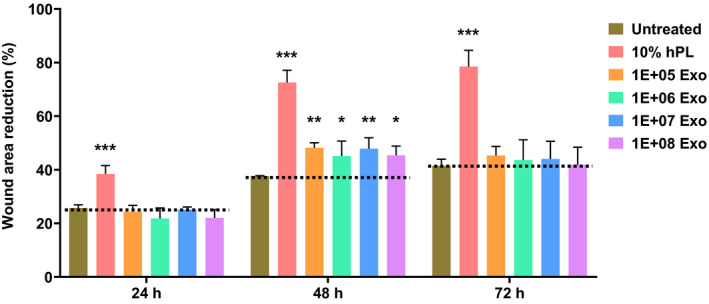
In vitro stimulation of fibroblasts migration by Exo_ucMSC_ assessed by wound healing assay. Confluent 3T3 cells (murine fibroblasts) were treated with 10 µg/mL mitomycin for 2 hours in serum‐free medium, prior to introducing a wound to the cell monolayer by scoring the cells with a pipette tip. Cells were washed and the medium replaced with that supplemented with 1% FBS, followed by treatment with 10% hPL (positive control) or varying amounts of Exo_ucMSC_. Images of the wound were taken at 0, 24, 48, and 72 hours post‐treatment, and the respective wound area was measured using ImageJ software. Rate of fibroblast migration is measured as the % difference in the wound area at each time point, relative to the wound area at 0 hour post‐treatment. Values are expressed as mean ± SD (n = 3). One‐way ANOVA with Dunnett's test for multiple comparisons was used for statistical analysis between all treatment groups with that of untreated group for each time point, respectively (*P < .05, **P < .01, ***P < .001)

Fibroblasts play a critical role in wound healing, where they migrate to the wound area and initiate the formation of granulation tissue, whereby new extracellular matrix proteins (ECM) is secreted to support and promote tissue remodeling.^43^, ^44^ Hence, the regenerative properties of Exo_ucMSC_ was investigated in terms of the ability of Exo_ucMSC_ EVs to promote fibroblast migration in an in vitro scratch assay to mimic wound infliction. hPL was used as positive control for fibroblast migration induction as previously reported.^45^ Treatment of the 3T3 murine fibroblasts with 10% hPL showed significantly higher fibroblast migration at 24, 48, and 72 hours post‐scratch compared to untreated cells (Figure 3). Following treatment with Exo_ucMSC_, a significantly higher fibroblast migration was also observed compared to untreated cells, but only after 48 hours post‐treatment, from a dose as low as 10^5^ particles. The effect was not dose dependent. Representative images of the migrating fibroblasts at 48 hours are shown in Supplementary Figure 4. Interestingly, all Exo_ucMSC_ doses tested did not result in a significantly higher fibroblast migration at 72 hours postscratch. In summary, Exo_ucMSC_ were able to stimulate fibroblast migration within 48 hours of wound infliction.

### 3.4 Exo_ucMSC_ stimulate fibroblast proliferation in vitro

**FIGURE 4:**
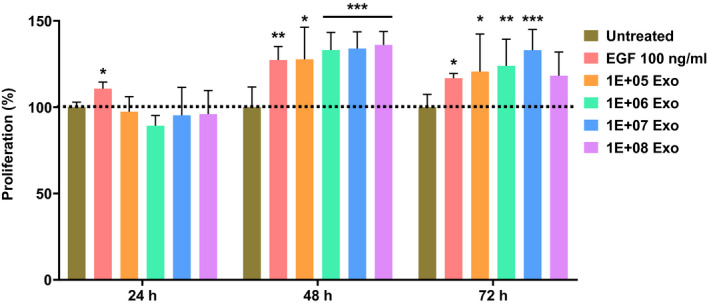
In vitro stimulation of fibroblasts proliferation by Exo_ucMSC_. 3T3 cells (murine fibroblasts) were treated with either 100 ng/mL EGF (positive control) or varying amounts of Exo_ucMSC_ for 24, 48, and 72 hours, after which the cells were subjected to an MTT assay. The OD_570nm_ values for each treatment group was normalized to that of the untreated group for each time point, and was taken as the measure of fibroblast proliferation. Values are expressed as mean ± SD (n = 12). One‐way ANOVA with Dunnett's test for multiple comparisons was used for statistical analysis between all treatment groups with that of untreated group for each time point, respectively (*P < .05, **P < .01, ***P < .001)

The proliferation of fibroblasts is also a key aspect in wound healing following migration, to allow a productive formation of granulation for proper wound healing.^43^, ^44^ Hence, the ability of regenerative properties of Exo_ucMSC_ in terms of stimulation of fibroblast proliferation was also investigated by means of MTT assay. Epidermal growth factor (EGF) was used as positive control for fibroblast proliferation induction as previously reported.^46^ Treatment of 3T3 cells with 100 ng/mL EGF showed significantly higher proliferation as compared to that of untreated cells at 24, 48, and 72 hours posttreatment. (Figure 4). Similar to the scratch assay above, 48 hours‐treatment with as low as 10^5^ Exo_ucMSC_ resulted in significantly higher fibroblast proliferation, to a similar extent to that by EGF. The same was observed with all the higher Exo_ucMSC_ doses tested, showing some dose‐dependent effect, but not statistically significant. Treatment with 10^5^‐10^7^ Exo_ucMSC_ doses also resulted in a significantly higher fibroblast proliferation at 72 hours, with a similar potency to that by EGF, except for 10^8^ Exo_ucMSC_. The former doses also showed a dose‐dependent trend, but not statistically significant. In summary, Exo_ucMSC_ were able to promote fibroblast proliferation from 48 hours up until 72 hours post‐treatment.

## 4 DISCUSSION

The Aggrewell system used in this work provides a straightforward and nonlabor‐intensive process for 3D culture of stem cells in a multi‐well culture plate format.^47^, ^48^ It enables the production of uniformly sized spheroids which can be easily harvested. Spheroid sizes can be tuned by controlling the seeding density or microwell size.^47^ A large number of spheroids per plate can be obtained, allowing facile scale‐up in a space‐efficient manner, which is an important criteria for clinical applications.^49^

Human platelet lysate (hPL) used for ucMSC culture in this work has been shown to contain various growth and soluble factors essential for cell culture.^50^ Hence, hPL serve as a viable alternative to FBS for preparation of clinical grade cell‐derived substances.^50^ Several studies have indeed reported successful expansion of MSC in hPL‐supplemented medium.^50^, ^51^, ^52^ However, similar to FBS, hPL also suffers from batch‐to‐batch variations, for both composition and concentrations of the various constituents, due to donor‐to‐donor variations and preparation methods.^51^ Such variations are likely to reflect on the biological responses of MSC or their derived exosomes.^51^ More importantly, hPL was reported to inherently contain exosomes.^53^ For this reason, a direct comparison of exosome yield and their biological responses could not be made between hPL and KO‐media 3D cultured ucMSC.

Only a limited number of studies to date have investigated spheroid formation by MSC in 3D culture. Studies on both adipose‐derived MSC (AD‐MSC) and ucMSC reported the spheroids to increase in size throughout the 3D culture.^38^, ^54^ These studies maintained the MSC spheroids culture for a much shorter duration than that in this work (7 and 9 days, respectively), and used FBS as medium supplementation. Our work is the first to investigate long‐term (24 days) spheroid formation of ucMSC in both hPL‐ and KO‐medium. ucMSC spheroids in this present work maintained their size in KO‐medium throughout culture, while spheroids in hPL‐medium gradually decreased in size (Figure 1). Given that the study on AD‐MSC mentioned above correlated the increasing spheroid size with increased cell survival marker (phosphorylated AKT) and lower levels of pro‐apoptotic proteins,^54^ we speculate the reduction in size of ucMSC spheroids in hPL medium to be associated with increasing levels of apoptosis throughout culture.

Therapeutic properties of exosomes were reported to be modulated by factors related to their culture condition such as cell source, oxygen tension, growth factor composition and physical microenvironment.^4^, ^7^, ^55^ Only two studies so far investigated the therapeutic effects of 3D culture‐derived Exo_ucMSC,_ reporting improved osteochondral regeneration^19^ as well as enhanced uptake and delivery of exogenously loaded siRNA against huntingtin.^56^ However, these studies used FBS in their 3D MSC culture, and employed different 3D culture setups (hollow fiber bioreactor system and micro‐carrier system, respectively). Another study investigated the therapeutic effect of exosomes derived from defined serum‐free 3D culture using MSC NutriStem XF medium. The study, however, used dental pulp‐derived MSC as a source of exosomes and used FBS in the initial 2D culture for expansion.^57^ Altogether, the difference in the growth factor composition, physical microenvironment and cell source in these studies made this current work the first to report on investigating the stimulation of fibroblast migration and proliferation—the key initial steps in wound healing by Exo_ucMSC_ obtained from defined serum‐free medium 3D ucMSC culture.

Exo_ucMSC_ have been reported to stimulate human dermal fibroblasts (HDF) migration and proliferation at 24 hours post‐treatment, of which the effect persisted and potentiated at 48 hours.^27^ Other studies using Exo_BM‐MSC_ and exosomes derived from AD‐MSC (Exo_AD‐MSC_) reported accelerated human fibroblast migration as early as 16 and 18 hours post‐treatment, respectively.^58^, ^59^ Studies using Exo_ucMSC_ and Exo_BM‐MSC_ treatment reported increased proliferation of HDF only at 72 hours post‐treatment, but the effects at earlier time points were not investigated.^30^, ^58^ In this work, murine 3T3 fibroblasts treated with Exo_ucMSC_ showed significantly accelerated migration only at 48 hours, while increased proliferation was observed at 48 and 72 hours post‐treatment (Figures 3 and 4). This work is also the first to investigate the effect of Exo_ucMSC_ on migration and proliferation of murine‐derived fibroblasts. This suggests that fibroblast migration and proliferation stimulation by Exo_ucMSC_ was more potent with earlier onset in human‐derived fibroblasts than those derived from mice. This is expected given that in the former, both the Exo_ucMSC_ and target cells are derived from humans. Interestingly, Exo_BM‐MSC_ was reported to promote equine fibroblast migration as early as 12 hours post‐treatment,^60^ potentially adding target cell species as another factor influencing MSC‐Exo properties alongside their culture conditions. Among biomolecules reported to be present within and delivered by MSC‐Exo that promote the migration and proliferation of fibroblasts and other cells such as keratinocytes include STAT3, Wnt4, and the long noncoding RNA (lncRNA) called metastasis‐associated lung adenocarcinoma transcript 1 (MALAT1).^58^, ^59^, ^61^ Target cell proliferation was also reported to be induced by MSC‐Exo‐mediated delivery and subsequent expression of the mRNA for growth factor receptors such as insulin like growth factor 1 receptor (IGF‐1R), which increases the sensitivity of the former to the trophic effect of IGF‐1.^62^ Future studies can investigate the effect of Exo_ucMSC_ and these associated molecules on the promotion of fibroblast migration and proliferation in vivo in preclinical murine models of cutaneous or vital organ tissue injury.

Excessive deposition of ECM by fibroblasts and myofibroblasts in the wound, as well as aberrant activation of the former into the latter contribute toward scar formation.^4^ This is undesirable as scar formation does not recover the original properties or function of the injured tissue.^63^ Treatment by Exo_AD‐MSC_ resulted in reduced scar formation in the healing wound partly by increased TGFβ3:TGFβ1 expression by fibroblasts.^8^ Although fibroblasts primarily play a role in ECM deposition, remodeling, and wound contraction,^64^ treatment by Exo_BM‐MSC_ increased their expression levels of growth factors such as hepatocyte growth factor (HGF).^58^ Both TGFβ3 and HGF were reported to be potent anti‐fibrotic factors, with the former being indispensable in scar‐free embryonic healing.^65^, ^66^, ^67^ The promotion of scar‐free wound healing in vitro and in vivo by Exo_ucMSC_ derived from defined serum‐free medium in this work by enhancing TGFβ3 and HGF expression would constitute another interesting and important future investigation.

3D culture of MSC was reported to augment their therapeutic effects.^37^ This benefit is also translated to exosomes, where Exo_ucMSC_ derived from 3D culture were reported to promote better osteochondral regeneration than that of their counterparts derived from 2D culture.^19^, ^68^ However, one study reported that the potency of Exo_BM‐MSC_ from 3D culture was not significantly different than that from 2D culture.^68^ Hence, it would be interesting to investigate if Exo_ucMSC_ in this work would show a higher potency than that derived from 2D culture in the same KO‐medium. Unfortunately, ucMSC were not viable in 2D culture in KO‐medium (Supplementary Figure 5), and this work is currently the first to report on this observation. This was attributed to the KO supplement lacking certain soluble molecules and factors that are present in serum,^69^ which was potentially compensated by the paracrine secretion of various growth factors by cells when cultured in 3D to form spheroids.^70^, ^71^ This is supported by reports on successful 2D culture of different MSCs in KO‐medium in the presence of feeder cells which helped to secrete growth factors into the media.^72^, ^73^ Feeder‐dependent cultures are not ideal in the context of deriving exosomes for use in therapy, as the conditioned medium (CM) would then also contain exosomes released by the feeder cells. It will then be difficult to attribute results from downstream functional studies to stem cell‐derived exosomes as no technique currently exists to separate exosomes from two different cell types from a single CM sample during isolation.

## CONFLICT OF INTERESTS

The authors have declared that no competing interests exist.

## AUTHORS’ CONTRIBUTIONS

F. N. Faruqu and K. T. Al‐Jamal designed the research; F. N. Faruqu, R. Liam‐Or, S. Zhou, and R. Nip performed the research; F. N. Faruqu, R. Liam‐Or, S. Zhou, and R. Nip analyzed the data; F. N. Faruqu prepared the manuscript; KT Al‐Jamal proof‐read the manuscript.

## Supporting information

Fig S1

Fig S2

Fig S3

Fig S4

Fig S5
