# Supplementary material for: Defined serum‐free three‐dimensional culture of umbilical cord‐derived mesenchymal stem cells yields exosomes that promote fibroblast proliferation and migration in vitro
Source: FASEB J. 2020 Dec 25;35(1):e21206. doi: 10.1096/fj.202001768RR (PMC7986687; doi:10.1096/fj.202001768RR)
Supplement: Supplementary file 2 — Fig S2 [file FSB2-35-0-s003.pdf]

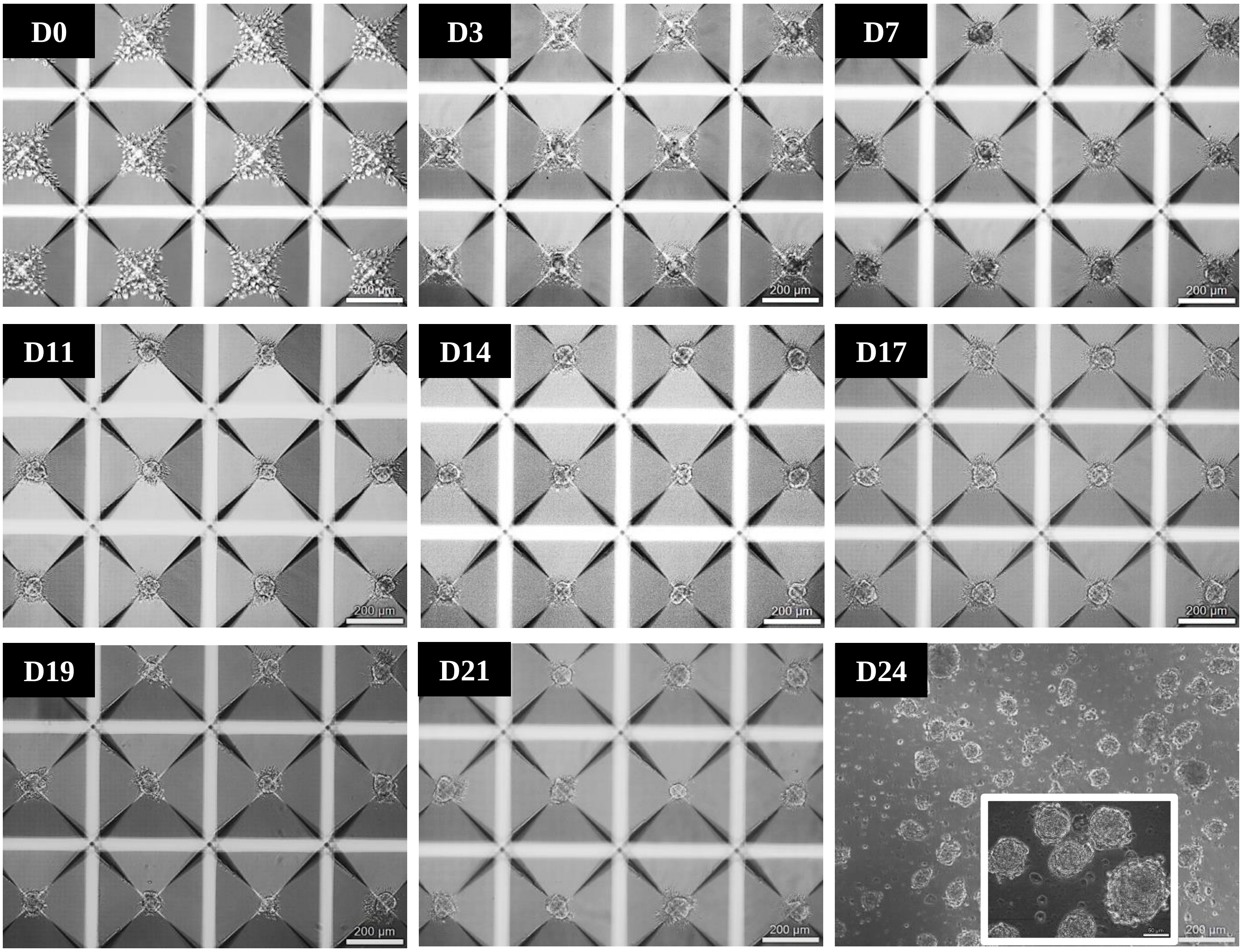

Supplementary Figure 2 ucMSC spheroid formation and maintenance in KO-medium in Aggrewell™ plate. Images show representative morphology of ucMSC forming spheroids in the microwell of the Aggrewell plates™ in KO-medium by bright field microscopy under 10X magnification throughout culture. Image on Day 24 shows harvested spheroids in suspension under 40X magnification, and the inset shows the spheroids under 40X magnification. ucMSC were seeded at a density of 1.2 x 10^5 cells/well (100 cells/microwell) of the Aggrewell™ plate.
