# Supplementary material for: Defined serum‐free three‐dimensional culture of umbilical cord‐derived mesenchymal stem cells yields exosomes that promote fibroblast proliferation and migration in vitro
Source: FASEB J. 2020 Dec 25;35(1):e21206. doi: 10.1096/fj.202001768RR (PMC7986687; doi:10.1096/fj.202001768RR)
Supplement: Supplementary file 3 — Fig S3 [file FSB2-35-0-s002.pdf]

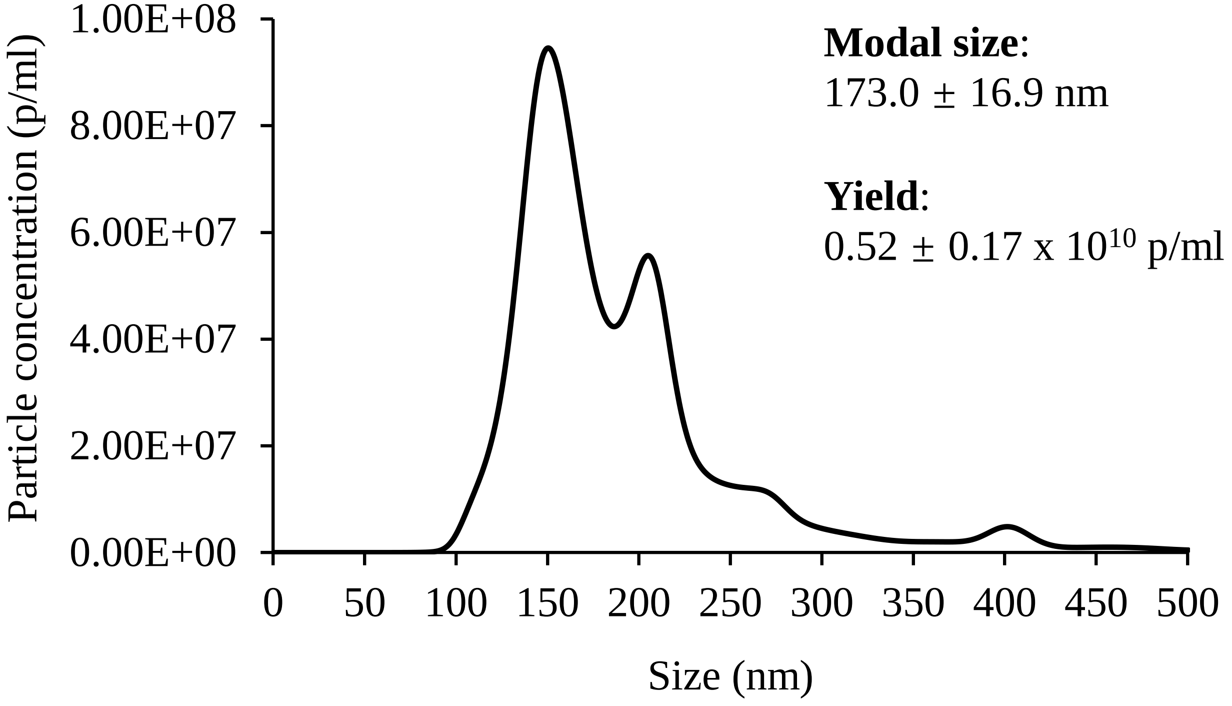

Supplementary Figure 3 Physicochemical characterisation of EVs isolated from unconditioned KO-medium (EV_{KO}). Freshly prepared KO-medium that has not been used for cell culture (unconditioned KO-medium) was subjected to the same exosome isolation protocol as conditioned KO-medium. Histogram shows results from nanoparticle tracking analysis (NTA) of the isolated EV_{KO}, resuspended in 400 µl PBS post-isolation. Both the yield and size of particles in EV_{KO} sample are significantly different than that in EV_{ucMSC} sample.
