# Supplementary material for: Defined serum‐free three‐dimensional culture of umbilical cord‐derived mesenchymal stem cells yields exosomes that promote fibroblast proliferation and migration in vitro
Source: FASEB J. 2020 Dec 25;35(1):e21206. doi: 10.1096/fj.202001768RR (PMC7986687; doi:10.1096/fj.202001768RR)
Supplement: Supplementary file 4 — Fig S4 [file FSB2-35-0-s005.pdf]

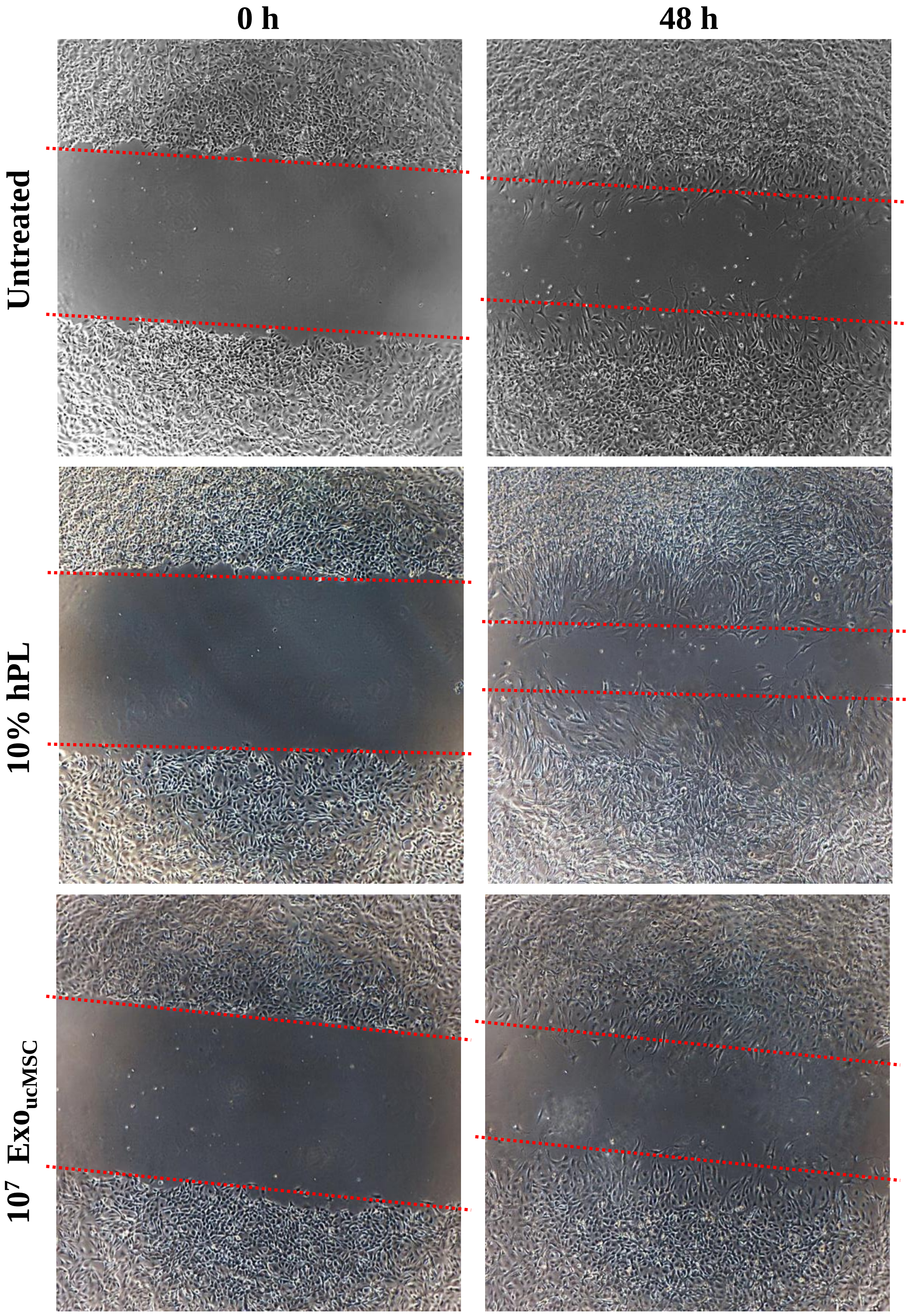

Supplementary Figure 4 ***In vitro*** **stimulation of fibroblasts migration by Exo$_{ucMSC}$ assessed by wound healing assay.** Confluent 3T3 cells (murine fibroblasts) were treated with 10 µg/ml mitomycin for 2 h in serum-free medium, prior to introducing a wound to the cell monolayer by scoring the cells with a pipette tip. Cells were washed and the medium replaced with that supplemented with 1% FBS. Images show representative wound area in fibroblasts which were untreated or treated with either 10% hPL (positive control) or 10^7 Exo$_{ucMSC}$ (representative dose) at 0 h and 48 h post-treatment. Rate of fibroblast migration is measured as the % difference in the wound area at each time point, relative to the wound area at 0 h post-treatment.
