# Supplementary material for: Defined serum‐free three‐dimensional culture of umbilical cord‐derived mesenchymal stem cells yields exosomes that promote fibroblast proliferation and migration in vitro
Source: FASEB J. 2020 Dec 25;35(1):e21206. doi: 10.1096/fj.202001768RR (PMC7986687; doi:10.1096/fj.202001768RR)
Supplement: Supplementary file 5 — Fig S5 [file FSB2-35-0-s001.pdf]

hPL

KO

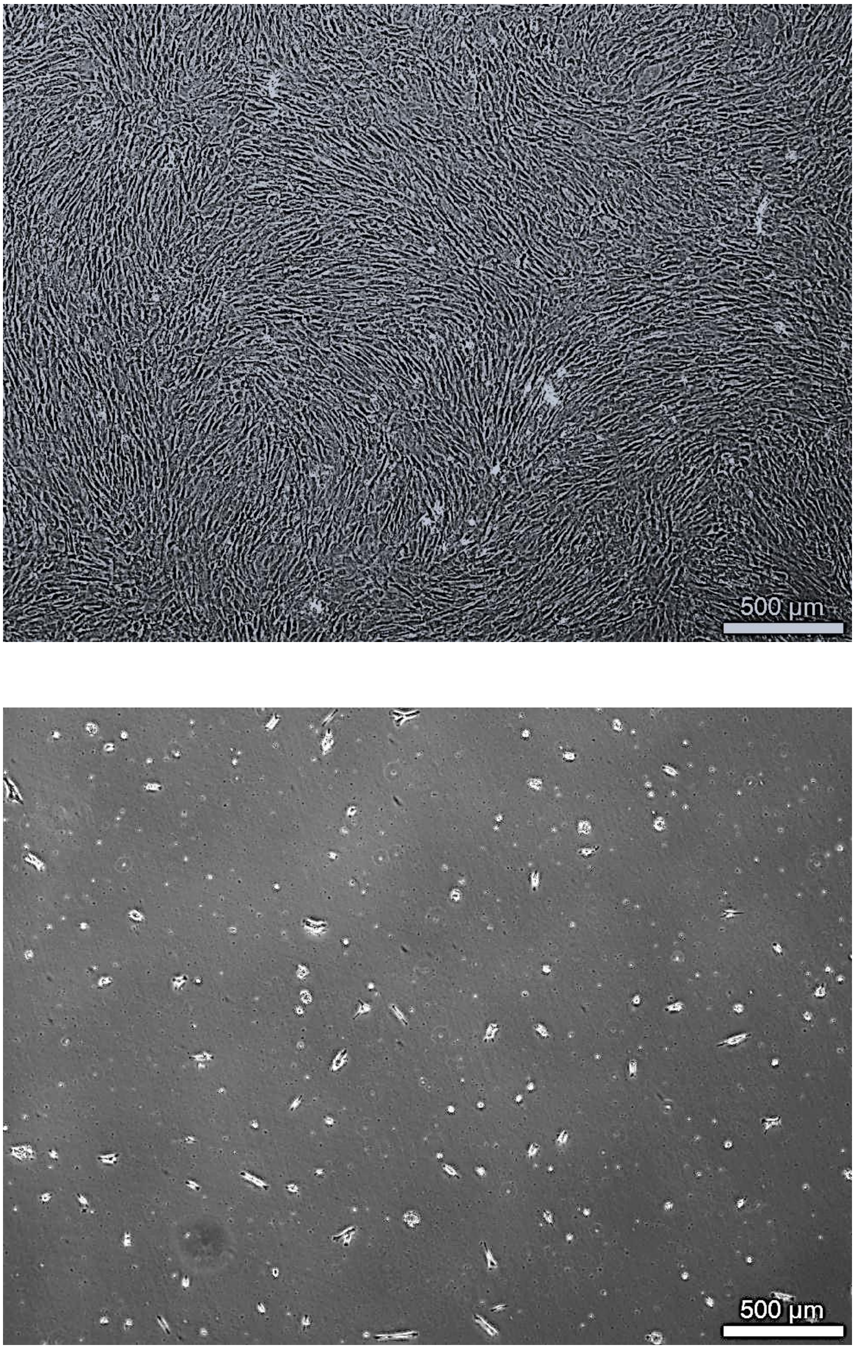

Supplementary Figure 5   2D culture of ucMSC in human platelet lysate (hPL)- and KO-medium. Images show representative morphology of ucMSC cultured in hPL- and KO- medium under bright field microscopy at 4X magnification. Cells were seeded at equal density (1:3 from a confluent 175 cm^2 flask) and images were taken after day 3 of culture.
